# Association between Microbiome-Related Human Genetic Variants and Fasting Plasma Glucose in a High-Cardiovascular-Risk Mediterranean Population

**DOI:** 10.3390/medicina58091238

**Published:** 2022-09-07

**Authors:** Eva M. Asensio, Carolina Ortega-Azorín, Rocío Barragán, Andrea Alvarez-Sala, José V. Sorlí, Eva C. Pascual, Rebeca Fernández-Carrión, Laura V. Villamil, Dolores Corella, Oscar Coltell

**Affiliations:** 1 Department of Preventive Medicine and Public Health, School of Medicine, University of Valencia, 46010 Valencia, Spain; 2 CIBER Fisiopatología de la Obesidad y Nutrición, Instituto de Salud Carlos III, 28029 Madrid, Spain; 3 Department of Computer Languages and Systems, Universitat Jaume I, 12071 Castellón, Spain; 4 Department of Phisiology, School of Medicine, University Antonio Nariño, Bogotá 111511, Colombia

**Keywords:** glucose, genetics, microbiome, Mendelian randomization, diet, Mediterranean

## Abstract

*Background and Objectives*: The gut microbiota has been increasingly recognized as a relevant factor associated with metabolic diseases. However, directly measuring the microbiota composition is a limiting factor for several studies. Therefore, using genetic variables as proxies for the microbiota composition is an important issue. Landmark microbiome–host genome-wide association studies (mbGWAS) have identified many SNPs associated with gut microbiota. Our aim was to analyze the association between relevant microbiome-related genetic variants (Mi-RSNPs) and fasting glucose and type 2 diabetes in a Mediterranean population, exploring the interaction with Mediterranean diet adherence. *Materials and Methods*: We performed a cross-sectional study in a high-cardiovascular-risk Mediterranean population (*n* = 1020), analyzing the association of Mi-RSNPs (from four published mbGWAS) with fasting glucose and type 2 diabetes. A single-variant approach was used for fitting fasting glucose and type 2 diabetes to a multivariable regression model. In addition, a Mendelian randomization analysis with multiple variants was performed as a sub-study. *Results*: We obtained several associations between Mi-RSNPs and fasting plasma glucose involving gut *Gammaproteobacteria_HB*, the order Rhizobiales, the genus *Rumminococcus* torques group, and the genus *Tyzzerella* as the top ranked. For type 2 diabetes, we also detected significant associations with Mi-RSNPs related to the order Rhizobiales, the family Desulfovibrionaceae, and the genus *Romboutsia*. In addition, some Mi-RSNPs and adherence to Mediterranean diet interactions were detected. Lastly, the formal Mendelian randomization analysis suggested combined effects. *Conclusions*: Although the use of Mi-RSNPs as proxies of the microbiome is still in its infancy, and although this is the first study analyzing such associations with fasting plasma glucose and type 2 diabetes in a Mediterranean population, some interesting associations, as well as modulations, with adherence to the Mediterranean diet were detected in these high-cardiovascular-risk subjects, eliciting new hypotheses.

## 1. Introduction

In the last decade, research into the effects of the gut microbiota (microorganisms)/microbiome (genome of these microbial populations) on health has intensified. Hundreds of papers have shown the enhancing or detrimental effects of the microbiota on health (depending on the type of microorganisms present in the intestine). These effects have been shown in a wide range of diseases, related to aging in general [1], as well as cardiovascular diseases [2,3], diabetes [4,5], obesity [5,6,7,8], neurodegenerative diseases [9,10,11], depression [12,13], and others [14,15,16,17].

Compared to the human genome, the gut microbiome is much more abundant (tens of times higher). It is estimated that there are more than 100 trillion microorganisms residing in our intestines [18]. Basically, the most studied microorganisms in the intestine have been bacteria, but there are also other types of microorganisms such as fungi, protozoa, and viruses, which require more research [19].

In humans, the composition of the gut microbiota is highly diverse and varies according to geographic origin, diet, age, drug use, and other factors related to lifestyle and health status [20,21,22]. The gut microbiota performs many host-friendly functions. It is widely documented that the microbiota is necessary to carry out functions related to the regulation of host immunity, protection against intestinal pathogens, strengthening gut integrity, functions related to the digestion and absorption of certain nutrients, and many others [21,22,23]. Thus, altered gut bacterial composition (so-called dysbiosis) has been associated with the pathogenesis of several diseases [22,23]. However, the composition of the microbiota is not easy to determine. The gut microbiota varies according to intestinal anatomical regions [22]. For this reason, it is important to standardize the collection of samples for microbiota determinations so that they are comparable between studies. In general, in epidemiological studies, feces are used for the analysis of the intestinal microbiota. The analysis of the microbiota in feces has gone from the use of very labor-intensive methods based on culture to other faster culture-independent approaches consisting of isolating microbial DNA and using high-throughput, low-cost sequencing methods [22,24]. These methods include 16S ribosomal RNA (rRNA) amplicon sequencing and shotgun sequencing [24]. Targeting of the bacterial 16S rRNA gene is the most used approach as it is faster and cheaper. However, 16S rRNA sequencing only profiles taxonomical composition (so-called metataxonomics), while shotgun metagenomic sequencing (so-called metagenomics) provides additional information about diverse species and functional annotations [24,25].

The Human Microbiome Project and Metagenomics of the Human Intestinal Tract (MetaHit) have provided important data on human-associated microbial composition [26,27]. These studies identified more than 2000 species isolated from humans, classified into 12 different phyla, of which 93.5% belonged to Proteobacteria, Firmicutes, Actinobacteria, and Bacteroidetes. The gut bacteria of healthy adults principally consist of six phyla: Firmicutes, Proteobacteria, Bacteroidetes, Actinobacteria, Fusobacteria and Verrucobacteria. Data from microbiome DNA sequencing allow for the quantification of taxa or genetic functions in stool samples. For example, hundreds of operating taxonomic units (OTUs) per individual can be characterized, and these OTUs can be collapsed into higher taxonomic levels throughout their phylogeny (genus, family, order, etc.). Likewise, amplicon sequence variants (ASVs) can be analyzed depending on the DNA sequencing method and the platform used [28,29]. Several studies have been carried out in humans using the 16S rRNA metataxonomics or the shotgun metagenomics methods to identify the microbiome profiles most associated with type 2 diabetes and insulin resistance [30,31,32,33,34,35,36,37]. Larsen et al. [35] in a pioneer and small study in Europeans reported that the phylum Firmicutes and class Clostridia were significantly reduced in diabetic subjects compared to nondiabetics. Furthermore, they observed that the ratios of Bacteroidetes to Firmicutes correlated directly with plasma glucose levels. Later, Zhang et al. [36] reported that the abundance of *Bacteroides* in type 2 diabetes subjects was only half that in nondiabetic groups. Moreover, they observed that butyrate-producing bacteria (e.g., *Akkermansia muciniphila* and *Faecalibacterium prausnitzii*) had a higher abundance in nondiabetics. This observation was consistently reported by subsequent studies; currently, one characteristic of the microbiome associated with type 2 diabetes is the moderate degree of gut microbial dysbiosis and the decrease in the abundance of some butyrate-producing bacteria. Other studies have reported an increase in various opportunistic pathogens, as well as other microbiome alterations [30,31,32,33,34,37]. However, consistency is still low, and more studies are needed in diverse populations to characterize the type 2 diabetes signature.

Unfortunately, the measurement of the microbiota is still difficult, and many epidemiological cohorts do not have stool analysis data. Therefore, other indirect measurements of the microbiota composition are being used more and more every day. These instrumental variables are microbiota-related human genes and are characterized by so-called single-nucleotide polymorphisms (Mi-RSNPs). Accumulating evidence is showing that host genetics may play a relevant role in gut microbial composition across species [38,39]. On this basis, many studies have been carried out to find out which genetic variants of the host are associated with the composition of the microbiota. Thus, several microbiome genome-wide association studies (mbGWAS) in different populations have reported dozens of host SNPs associated with the gut microbiome composition at the taxon, family, genus, or species level [40,41,42,43,44,45,46,47,48,49,50,51]. The use of these SNPs as Mi-RSNPs allows us to undertake so-called Mendelian randomization studies [52,53]. Mendelian randomization uses gene variants, which are fixed at conception, to support causal inferences about the effect of modifiable exposures (or risk factors) that can overcome some types of confounding and/or reverse causality [54,55]. However, we must be cautious when stating that there are causal associations as Mendelian randomization studies (one-sample or two-sample) [56] also have many drawbacks and limitations [57]. Moreover, population stratification and geographical origin are relevant factors influencing bias in genetic associations [58], and more studies in understudied populations are needed. Therefore, our aims were (1) to analyze the association between relevant Mi-RSNPs and fasting plasma glucose and type 2 diabetes in a Mediterranean population, exploring the interaction with Mediterranean diet adherence, and (2) to undertake a formal two-sample Mendelian randomization study based on a published mbGWAS predicting microbiota composition in a European population (sample 1) and testing the effect of multiple instruments on fasting glucose in the Mediterranean population (sample 2).

## 2. Materials and Methods

### 2.1. Participants and Study Design

First, a cross-sectional study on 1020 elderly high-cardiovascular-risk Mediterranean subjects was undertaken. Second, a two-sample Mendelian randomization was performed. The recruited white European subjects were participants in the PREDIMED (Prevención con Dieta Mediterránea) Valencia study [59], from the Valencia field center, located on the Mediterranean coast of the Iberian Peninsula (Spain). These participants were recruited in primary healthcare centers, with the following inclusion criteria: elderly (between 55 and 80 years old for men and between 60 and 80 years old for women) and having a high cardiovascular risk, even though they were free of cardiovascular disease at baseline and had plasma fasting glucose determined, as well as successful DNA high-density DNA genotyping for this study (in addition to the other covariates). The specific inclusion criteria were to have either type 2 diabetes or three or more major cardiovascular risk factors, out of the following: current smoking, hypertension, dyslipidemia, body mass index (BMI) ≥ 25 kg/m^2^, and /or family history of premature cardiovascular disease [60]. Participants provided written informed consent, and the study was conducted according to the guidelines of the Declaration of Helsinki and approved by the Human Research Ethics Committee of Valencia University, Valencia (ethical approval code H1491427097983, 8 May 2017).

Summary data of the association between gut microbiota composition and genetic variants were obtained from four published mbGWAS [43,45,46,51]. For the two-sample Mendelian randomization analysis (referring to the application of Mendelian randomization methods to association results estimated in two nonoverlapping sets of individuals) we used, as one sample, the summary level data obtained in the published study by Qin et al. [51] on a large-scale population-based cohort of 5959 individuals with matched gut microbial metagenomes from the FINRISK study. FINRISK is a large Finnish population survey on risk factors in chronic diseases, including subjects aged between 25 and 74 years from six geographical areas of Finland. The study protocol of this study was approved by the Coordinating Ethical Committee of the Helsinki and Uusimaa Hospital District (Ref. 558/E3/2001). For analyzing available summary data from this and from the other already published studies [43,45,46], no additional permission was required. The second sample was our Mediterranean population.

### 2.2. Baseline Anthropometric, Clinical, Biochemical, and Lifestyle Variables

In our Mediterranean population, we assessed demographic variables, cardiovascular risk factors, and clinical and lifestyle variables by validated questionnaires as previously reported [59]. Anthropometric variables and blood pressure were determined at baseline by trained staff. Weight and height were measured with light clothing and no shoes with calibrated scales and a wall-mounted stadiometer, respectively. BMI was calculated as the weight (in kg) divided by the height (in m^2^). Blood samples were collected after a 12 h overnight fast. Fasting glucose, total cholesterol, triglycerides, and HDL cholesterol (HDL-C), were measured using standard enzymatic automated methods as previously described [61]. Type 2 diabetes was defined as previously reported [60]. We analyzed adherence to the Mediterranean diet pattern in our Mediterranean population using the validated 14-item scale [62], also administered at baseline. Detailed items of that scale with their response options have been published elsewhere [60,62]. Briefly, the scale consisted of 12 questions on food consumption frequency and two questions on food intake habits considered characteristic of the Mediterranean diet. Each question was scored 0 or 1. The final score ranged from 0 to 14. One point was given for reaching the adherence to the Mediterranean diet recommendation for each item in terms of amount and frequency (olive oil, preferring white meat over red meat, two or more servings of vegetables/day; three or more pieces of fruit/day; <1 serving of red meat or sausages/day; <1 serving of animal fat/day; <1 cup of sugar-sweetened beverages/day; moderate consumption of red wine; three or more servings of pulses/week; three or more servings of fish/week; fewer than two commercial pastries/week; three or more servings of nuts/week; two or more servings/week of “sofrito” (traditional sauce of tomatoes, garlic, onion, or leeks sautéed in olive oil). If the condition was not met, 0 points were recorded for the category. A higher score indicated a greater the adherence to the Mediterranean diet. The degree of adherence was later dichotomized into low or high adherence depending on the population mean (9 points).

### 2.3. DNA Isolation, Genotyping and GWAS in This Mediterranean Population

Genomic DNA was isolated from blood. The quantity of double-stranded DNA was measured using PicoGreen (Invitrogen Corporation, Carlsbad, CA, USA), and high-density genotyping at the genome-wide level using the Infinium OmniExpress-24 BeadChip genotyping array (v1.0 and v1.1) (Illumina Inc., San Diego, CA, USA) was undertaken. This array captures approximately 720,000 markers (the number varies depending on the version: 730,000 for v.1.0 and 716,000 for v.1.1, with 699,221 markers that are common to both versions of the array). Genome-wide genotyping was performed at the University of Valencia according to the manufacturer’s protocol with appropriate quality standards as previously reported [59]. Data cleaning was performed using standard analysis pipelines implemented in Python programming language using the Numpy library modules combined with PLINK [63,64]. SNPs not mapped on autosomal chromosomes were filtered out. In addition, SNPs with a minor allele frequency (MAF) < 0.01, those that deviated from the expected Hardy–Weinberg equilibrium (*p* < 1.0 × 10^−4^), and SNPs with a low call rate (<90%) were removed. The overall call rate in these subjects exceeded 99% genotyping. The minor allele frequency (MAF) was obtained for each SNP in this population. Using genome-wide genotyping, we undertook various GWASs (crude and adjusted for several confounders) to identify which gene variants were most associated with plasma fasting glucose and type 2 diabetes. Additive genetic models were fitted. General linear models were used for fasting glucose as the dependent variable, and regression coefficients and SE were estimated. Models were sequentially adjusted for sex, age, type 2 diabetes, BMI and diabetes medication. Beta coefficients for the minor allele were obtained. These analyses were performed on the whole population and stratified by type 2 diabetes when indicated. Moreover, the statistical significance of the genome-wide SNP × Mediterranean diet pattern (low vs high) interaction term was computed, as well as the interaction term SNPs × sex. With type 2 diabetes as the dependent variable, we used logistic regression analysis (unadjusted and adjusted for the indicated potential confounders). Odds ratios (ORs) and the corresponding 95% confidence intervals (CI) were estimated. Although all the participants were white Caucasians, and no ethnicity bias or population stratification was expected, we checked this potential influence by calculating the genomic inflation factor (lambda coefficient), and it was equal to 1, indicating no genomic inflation. The quantile–quantile plots (Q–Q plots) comparing the expected and observed *p*-values were performed in the R statistical environment [65].

Later, from these summary statistics we selected the SNPs corresponding to the microbiome-related genes (the Mi-RSNPs) identified from the previously selected mbGWASs [43,44,45,46,51] and extracted the association coefficients (beta, SE, and *p*-values) for further analyses. Only the results corresponding to the selected Mi-RSNPs were included in this paper.

### 2.4. Selection of the Mi-RSNPs from Previously Published mbGWAS and Statistical Analysis

Summary data of the association between gut microbiota composition and genetic variants were obtained from four published mbGWASs [43,45,46,51]. These studies were so-called STD1 [45], STD2 [46], STD3 [51], and STD4 [43] for the purposes of data analysis and tables in this manuscript. Although these studies listed several SNPs as Mi-RSNPs in the reported results, we selected SNPs on the basis of the study *p*-value (*p* < 5 × 10^−8^ as the preferred *p*-value or *p* < 1 × 10^−5^ when very few SNPs reached the GWAS level of significance) and the MAF of the corresponding SNP in the Mediterranean population (MAF > 0.05). We also considered the coverage of our Illumina Human OmniExpress Array, due to the fact that no imputations were carried out in our Mediterranean population. STD1 was undertaken by Hughes et al. [45] in Germany. The authors analyzed fecal-derived 16S rRNA gene sequences and host genotype data from the Flemish Gut Flora Project (*n* = 2223) and two German cohorts (FoCus, *n* = 950, PopGen *n* = 717), and identified Mi-RSNPs involving multiple microbial traits. STD2 was undertaken by Kurilshikov et al. [46], analyzing the effect of host genetics on gut microbiome composition (16S fecal microbiome data) from 18,340 individuals (24 cohorts). STD3, carried out by Qin et al. [51], analyzed a population-based cohort consisting of 5959 participants in the FINRISK study. Genome-wide genotyping and metagenome data from stools were obtained. STD4, undertaken by Davenport et al. [43], examined the association host genotypes with the relative abundance of fecal bacterial taxa in the Hutterites (*n* = 186 samples). Table S1 shows the list of the selected Mi-RSNPs (and the microbial trait) extracted from these studies [43,45,46,51] according to the abovementioned criteria and used in the statistical associations with fasting glucose and type 2 diabetes in our Mediterranean population. Analyses were undertaken for the whole population and stratified by type 2 diabetes when indicated. All tests were two-tailed, and *p*-values <0.05 were considered statistically significant for these associations.

### 2.5. Two-Sample Mendelian Randomization Analysis for Microbiome Effects on Fasting Glucose

For the two-sample Mendelian randomization analysis, we used, as population 1 (for the association between the instrumental variables and the exposure), the summary-level data obtained in the mbGWAS undertaken in 5959 participants from the FINRISK study [51] (STD3). Taxonomic profiling in the FINRISK study was performed according to the taxonomic nomenclature updated in the Genome Taxonomy Database (GTDB) release 89, and gut microbial composition was represented as the relative abundance of taxa. For each metagenome at phylum, class, order, family, genus, and species level, the relative abundance was computed as the proportion of reads assigned to the clade among the total reads [51]. The second sample was our Mediterranean population, and we tested the association between the instrumental variables and the outcome (plasma fasting glucose; expressed in mg/dL per allele). Population 1 was selected because information related to the effect allele, regression coefficient (beta estimations per effective allele), and SE were uniformly presented in tables. In addition, the authors used the Human OmniExpress array for genotyping, as we did in our Mediterranean population. No information from the other studies was combined in the Mendelian randomization approach to minimize the population heterogeneity bias and the methodological differences in the coefficient estimations. We selected the Mi-RSNPs as instrumental variables from Table S1 on the basis of the strength of the association (*p*-value and F statistics) between the Mi-RSNPs and the microbiome trait in population 1, as well as those top ranked in population 2 (excluding SNPs with MAF <0.1 in population 2), excluding previously reported pleiotropic SNPs [66]. Exposure and outcome were harmonized to ensure that both datasets were identically coded regarding the effect allele to reduce issues with palindromic variants. First, the effect allele and the corresponding beta were oriented to the risk-increasing alleles in dataset1 corresponding to the exposure (microbiome). Then, the same allele was considered to compute the beta corresponding to the outcome in dataset2 [67]. Different Mendelian randomization methods were used to estimate the so-called “causal” effect: the simple median [68], the weighted median [69], the inverse variance weighted method (IVW) [70], and the Mendelian randomization Egger method (MR-Egger) [71,72]. Additionally, robust methods in Mendelian randomization via penalization of heterogeneous causal estimates were computed [73]. Heterogeneity statistics and sensitivity analyses were performed. Statistical analyses were conducted using the R package “TwoSampleMR” [73,74,75].

## 3. Results

### 3.1. General Characteristics of the Participants

The demographic, anthropometric, clinical, biochemical, and lifestyle characteristics of the study participants at baseline are presented in Table 1. We analyzed 1020 subjects (371 men and 649 women). They consisted of elderly men and women from a Mediterranean population (mean age 67.9 ± 0.2 years) at a high cardiovascular risk.

BMI was high (mean 30.6 ± 0.2 kg/m^2^), with differences per sex. Prevalence of type 2 diabetes was 46.5%, being higher in men than in women (*p* < 0.05). Diabetes medications included insulin analogs (15%) and oral glucose-lowering drugs. Mean adherence to the Mediterranean diet according to the P-14 scale was 8.5 ± 0.1 points. No statistically significant sex-specific differences in the Mediterranean diet adherence were detected per sex (*p* = 0.147).

### 3.2. Association between the Microbiome and Fasting Plasma Glucose

First, we investigated the association between the microbiome and fasting plasma glucose in the whole population using the selected Mi-RSNPs (*n* = 209, listed in Table S1) as proxies involving one phylum, seven classes, seven orders, five families, 69 genera, and 11 species. First, we fitted a crude model (model 1), and we later sequentially adjusted for additional covariates (sex, age, type 2 diabetes, BMI, and diabetes medication). We observed a high consistency in the top-ranked SNPs across the different models. Table 2 shows the associations (regression coefficient and *p*-values) between the selected Mi-RSNPs and fasting plasma glucose in the whole population (*n* = 1020) for the most significant SNPs (top-ranked) in a model adjusted for sex, age, type 2 diabetes, BMI, and diabetes medication.

Very few Mi-RSNPs reached the nominal statistical significance (rs9569095, rs17103336, rs11940694, rs10498633, rs2269706, rs17085775, and rs10752747). The hit was the Mi-RSNP rs9569095 located at LOC105370213, in chromosome 13. This gene is a still uncharacterized RNA gene and is affiliated with the noncoding (nc) RNA class. The SNP was reported in STD1 (45) to be associated with class *Gammaproteobacteria*_*HB*. In our Mediterranean population, we found that the minor allele of this SNP was associated with less fasting glucose (*p* = 0.00099). The other hits at *p* < 0.01 were Mi-RSNPs previously related to the order Rhizobiales and the genus *Rumminococcus* torques group. Lastly, we detected at *p* = 0.0363 the Mi-RSNP rs10752747 in the membrane metalloendopeptidase like 1 (MMEL1) gene, previously related to abundance of the genus *Tyzzerella* (subgroup 3). Considering the potential interest for subsequent meta-analysis, in Table S2, we present the results for the other Mi-RSNPs not reaching a statistically significant association (from *p* > 1.2090 to *p* ≤ 0.3350) with fasting plasma glucose in the whole population.

In addition, we studied the association between the selected Mi-RSNPs and fasting plasma glucose concentrations in nondiabetic subjects (*n* = 546). Table S3 shows the results for the top-ranked SNPs. The hit was the rs6890044 in chromosome 5 (intergenic). This Mi-RSNP was related to *Massiliomicrobiota* species. The second top-ranked SNP was the rs910633, also intergenic in chromosome 1, related to the genus *Faecalibacterium*. Interestingly, the rs17103336 in the BTB domain-containing 16 (BTBD16) gene, a proxy for the order Rhizobiales and previously identified as top ranked for the whole population (Table 2), reached statistical significance as the third hit in nondiabetic subjects.

### 3.3. Association between the Microbiome and Type 2 Diabetes Prevalence

Furthermore, we analyzed the association between the Mi-RSNPs (listed in Table S1) and type 2 diabetes prevalence in this Mediterranean population. Table 3 shows the obtained results listing the top-ranked SNPs sorted in ascending order in terms of their *p*-value.

In the model adjusted for sex, age, and BMI, the hit Mi-RSNP was rs17551124 (*p* = 0.009) intergenic in chromosome 10, a proxy for the order Rhizobiales. We also detected significant associations at the nominal *p*-value with the proxies for the family Desulfovibrionaceae (rs17063777; intergenic chromosome 4) and rs10091895 in the *CUB* and *Sushi multiple domains 1* (CSMD1), a proxy for the genus *Romboutsia*.

### 3.4. Interactions between the Mi-RSNPs and Adherence to the Mediterranean Diet in Determining Fasting Plasma Glucose

The mbGWASs from which the previously analyzed Mi-SNPs were obtained were carried out fundamentally in non-Mediterranean populations [43,45,46,51]. Although, in these previous GWAS, the degree of adherence to the Mediterranean diet was not characterized, the influence of milk consumption on the effect of SNPs close to the lactase gene (LCT) was reported. Therefore, we considered it interesting to explore whether the level of adherence to the Mediterranean diet in our population could modulate the associations of the Mi-RSNPs on plasma fasting glucose. We considered this approach as an exploratory study due to the fact that the sample size has to be larger to better characterize gene–diet interactions.

Two levels of adherence to the Mediterranean diet were considered (low adherence and high adherence) on the basis of the P-14 scale described in Section 2. Table 4 shows the top-ranked Mi-SNPs ordered by the statistical significance of the interaction term between adherence to the Mediterranean diet and the SNP on plasma fasting glucose.

Regression coefficients and SE for the effect in subjects with low (strata 1) and high (strata 2) adherence are presented. The most significant *p*-value (*p* = 0.006) for an Mi-RSNP and the Mediterranean diet interaction was detected with rs2716882, intergenic in chromosome 17.

This SNP is a proxy for the genus *Sporacetigenium*. According to this interaction, the effect of this SNP on plasma fasting glucose differed depending on the adherence to the Mediterranean diet. When adherence was low (strata 1), the minor allele was associated with increased fasting glucose (beta = 12.4 mg/dL), whereas when the adherence was high, the minor allele was not associated with such an increase (beta = −0.45 mg/dL). We also obtained statistically significant interactions with nine other Mi-RSNPs (rs16833405, rs7580217, rs6717477, rs6546314, rs7104796, rs10520163, rs7773795, rs11141878, and rs12597384) involving several genera (*Acinetobacter*, *Desulfovibrio*, *Allisonella*, etc.), suggesting a relevant role of the dietary pattern when analyzing the host–microbiome associations.

### 3.5. Interactions between the Mi-RSNPs and Sex in Determining Fasting Plasma Glucose

Another potential modifier of the association effects between the microbiota and the outcome was sex. In our study analyzing a Mediterranean population, we had the advantage of being able perform sex-specific analyses, since, instead of using summary data only, we undertook genetic studies in our population. For this reason, we had more flexibility to carry out analyses stratified by sex. However, instead of delving into these stratified analyses in which the sample size was smaller and the number of comparisons made also increased, we chose to perform an exploratory analysis examining gene–sex interactions. Table S4 shows the top-ranked Mi-SNPs ordered by the statistical significance of the interaction term between sex and the corresponding SNP on plasma fasting glucose. Regression coefficients and SE for the effect in men (strata 1) and women (strata 2) are presented. The most significant *p*-value (*p* = 0.0019) for an Mi-RSNP and sex interaction was detected with rs367480 SNP located in the solute carrier family 22 member 18 (SLC22A18) gene, a proxy for the order Gastranaerophilales. This sex–gene interaction suggested opposite effects in men and women. We also detected statistically significant sex–gene interactions at the nominal level with 12 other Mi-RSNPs (rs7129903, rs13132148, rs11865270, rs9401713, rs12530266, rs1490359, rs4479964, rs11940694, rs1699103, rs12597384, rs639648, and rs4756282) related to the Enterobacteriaceae family and to several genera (*Blauntia*, *Clostridium*, *Dialister*, *Odoribacter*, etc.). This sex–gene interaction results support a more detailed sex-specific analysis in the ongoing and future research in the field.

### 3.6. Mendelian Randomization Sub-Study including Multiple Mi-RSNPs in Determining Plasma Fasting Glucose

**Figure 1:**
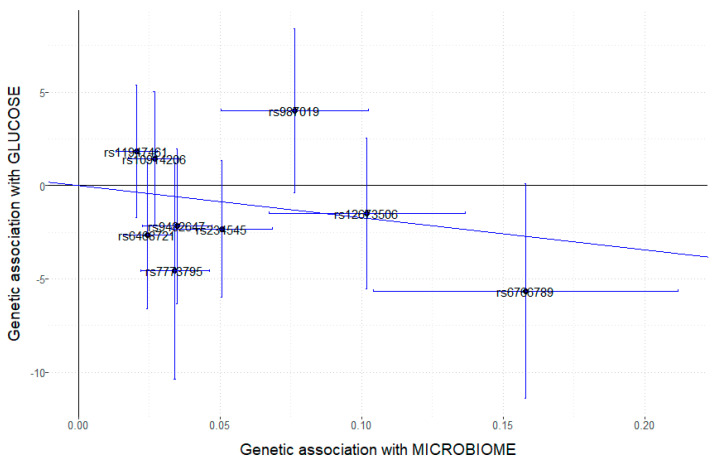
Scatter plot of genetic associations with gut microbiome against the genetic association with outcome (fasting plasma glucose for the nine preselected instruments—indicated SNPs). SNP: Single nucleotide polymorphism.

Above, we analyzed the Mi-RSNPs separately. However, the microbiota can exert its effects jointly, such that it is a combination of taxa that enhances the favorable or detrimental effects on the studied outcome. Due to the fact that the previously selected Mi-RSNPs (Table S1) came from several studies carried out in different populations [43,45,46,51] and with different methodologies, to perform the Mendelian randomization analysis, we preferred to consider only a single mbGWAS carried out uniformly in a European population, the FINRISK study [51]. From this European population (*n* = 5959), we selected genetic instruments as detailed in Section 2 and obtained the instrument–exposure associations. Then, we performed a two-sample Mendelian randomization study [67] using our Mediterranean population as the second population (*n* = 1020) to test the instrument–outcome associations. We preselected the first (*n* = 12) top-ranked Mi-RSNPs from the FINRISK study on fasting glucose in our population and tested if these Mi-RSNPs were a good instrument according to Mendelian randomization core assumptions [52,58]. The *p*-value for the associations between each of them with the exposure (microbial trait) was very significant (ranging from 4 × 10^−8^ to 8 × 10^−9^), and the F statistics were >10. In addition, genetic variants would be excluded if the MAF was less than 0.1. We detected and excluded two SNPs with MAF <0.10. Therefore, the first assumption, relating to the strength of the association with the exposure, was confirmed. The second Mendelian randomization assumption was related with pleiotropy, and we excluded the MCM6 SNP, as previously reported [66]. Later, the MR-Egger regression slope was tested to confirm the absence of pleiotropy. Thus, nine Mi-RSNPs were preselected as good instrumental variables for the Mendelian randomization study with multiple instruments. Table S5 shows the nine preselected instruments, the related microbial trait and the coefficients for the two populations after the required harmonization, as detailed in Section 2. Different Mendelian randomization methods were used to estimate the so-called “causal” effect, as detailed in Section 2. Figure 1 shows the scatter plot of genetic associations with gut microbiome against the genetic association with outcome (fasting plasma glucose) for the nine preselected instruments (indicated SNPs).

Table 5 shows the Mendelian randomization estimates for each method of the so-called “causal” effect of the gut microbiome for the nine preselected instruments (Mi-RSNPs) on plasma fasting glucose concentrations.

The simple median (*p* = 0.065), weighted median (*p* = 0.099), and IVW (*p* = 0.233) showed no statistically significant results. Only the penalized IVM (*p* = 0.038) and the penalized robust IVM (*p* = 0.002) found significant evidence for the association. In these methods, penalization of heterogeneous causal estimates was computed. MR-Egger regression further suggested no horizontal pleiotropy (slope: 0.351; *p* = 0.488).

**Figure 2:**
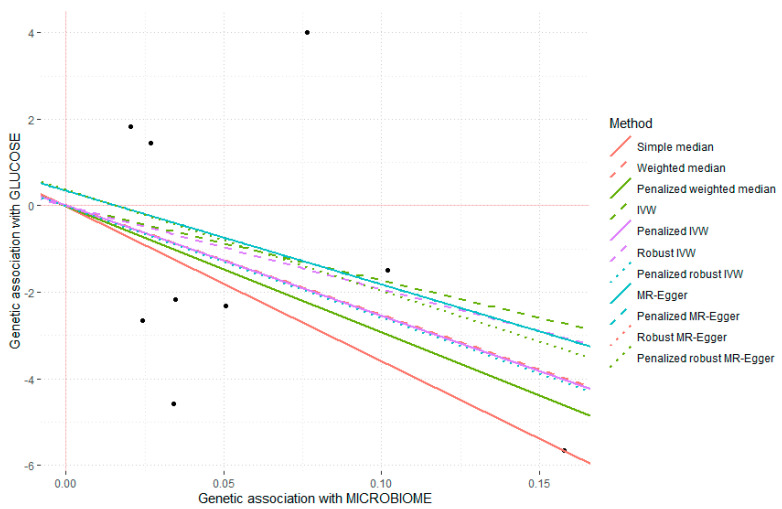
Scatter plot and regression lines of genetic associations with gut microbiome (nine SNPs) against the genetic association with fasting plasma glucose. The slope of the lines represents the so-called “causal” association, and each method has a different line. SNPs: Single nucleotide polymorphisms.

Figure 2 shows the scatter plot and the regression lines of genetic associations with gut microbiome against genetic associations with fasting glucose.

The slope of the lines represents the so-called “causal” association for each method indicated in the legends. Heterogeneity statistics and sensitivity analyses were performed. The MR-Egger heterogeneity test (*p* = 0.063) suggested some heterogeneity.

**Figure 3:**
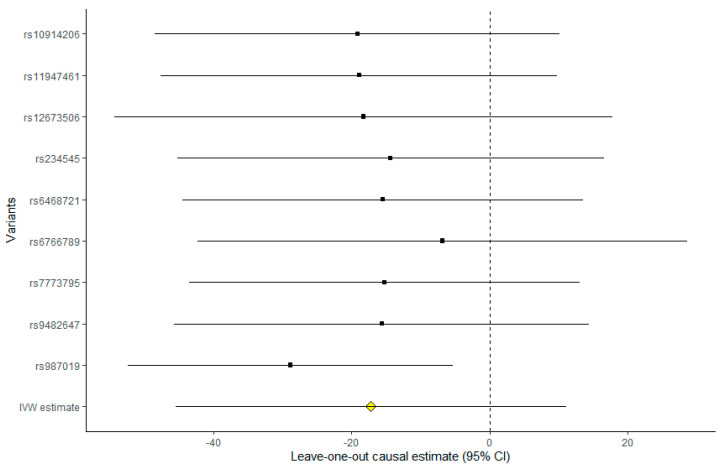
Leave-one-out sensitivity analysis for the association using the nine SNPs. Each point and error line represents the MR analysis (using IVW) excluding that particular SNP. The overall analysis including all SNPs is also shown for comparison. SNPs: Single nucleotide polymorphisms.

The leave-one-out sensitivity analysis (Figure 3) was performed to ascertain if the global Mendelian randomization association was influenced by a single SNP. Each point and the error lines for each SNP represents the Mendelian randomization association (using the IVW method) excluding that particular SNP. The overall analysis including all SNPs is also shown for comparison. We detected that the rs987019 SNP was an influential SNP.

This SNP was detected as a potential outlier in Figure 1, due to the fact that the association between the increases in the exposure level was associated with less fasting glucose for many SNPs, except the rs987019 SNP. This Mi-RSNP was related to increased levels of *Romboutsia ilealis* [51], and, although the evidence is not consistent, some studies related increased abundance of *Romboutsia* to an increased risk of insulin resistance and obesity [76,77]. Hence, we recalculated the “causal” effect estimate after removing the rs987019 SNP considered as an outlier. The Mendelian randomization analysis with the remaining eight instrumental variables showed statistically significant results using the simple median method (*p* = 0.031) and using all the IVW methods: IVW (*p* = 0.016), penalized IVW (*p* = 0.016), robust IVW (*p* < 0.001), and penalized robust IVW (*p* < 0.001). The heterogeneity MR-Egger test was improved (*p* = 0.340), and no horizontal pleiotropy was detected (slope MR-Egger = 0.310; *p* = 0.888).

**Figure 4:**
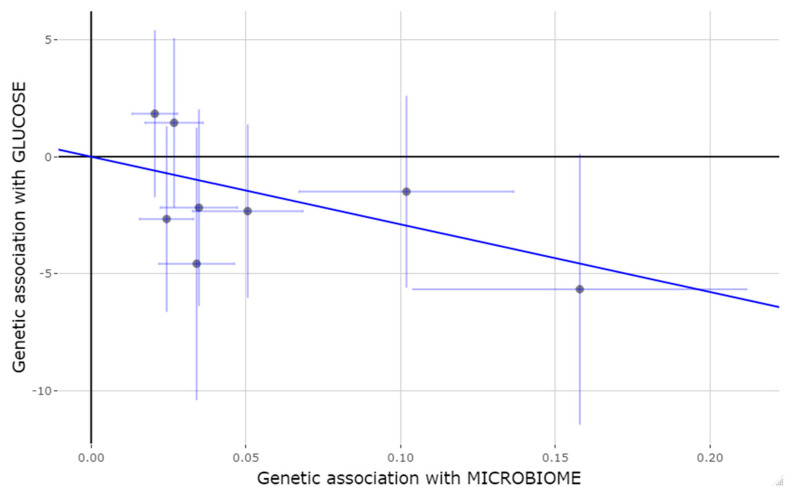
Mendelian randomization estimates for the association with fasting plasma glucose for the eight selected instruments. Individual SNPs and global association using the IVW method. SNPs: Single nucleotide polymorphisms.

Figure 4 shows the so-called “causal” estimates for the association between the microbiome and fasting glucose using the eight finally selected instruments and presents estimations for the individual SNPs, as well as the global association using the IVM method. According to this estimation, an increasing number of effect alleles for these SNPs was related to a rise in the corresponding taxa abundance related to these SNPs, and this increase was associated with less fasting plasma glucose in the host (IVM method: −28.8; 95%CI: −52.4 to −5.4; *p* = 0.016).

## 4. Discussion

To our knowledge, this paper represents the first study performed to examine the interplay among host genetics, the gut microbiota, and fasting glucose and type 2 diabetes using Mi-RSNPs as proxies for microbiome composition in a high-cardiovascular-risk Mediterranean population. In the last decade, the number of studies analyzing the association between the composition of gut microbiota and different health phenotypes has increased exponentially, both in animal models and in humans [78,79]. Every day, new results are published on the influence of the gut microbiota, fundamentally bacterial, in different phenotypes of cardiometabolic diseases, as well as other diseases [1,2,3,4,5,6,7,8,9,10,11,12,13,14,15,16,17]. Therefore, it is not surprising that researchers from different areas of health are interested in incorporating the influence of the intestinal microbiota in their analyses from a multifactorial point of view. However, the study of the gut microbiota is complex and requires the collection of feces from the study participants. In addition, there are different platforms and analysis methodologies that add complexity to the comparison of results [24,25,28,29]. For this reason, the possibility of using some genomic markers of the host as indicator variables of the composition of the intestinal microbiota is arousing great interest in the scientific community. However, these studies are still very preliminary and need further extension and replication in different populations.

Some studies in several species (including *Drosophila*, *Caenorhabditis elegans*, mice, and other animals) have shown significant heritability, specific to different microbial families and genera [79]. In humans, the influence of host genetics on the microbiome was less recognized in early studies; however, currently, several landmark mbGWASs have identified many human genetic variants associated with gut microbiota [41,42,43,45,46,47,48,49,50,51,80]. These variants, so-called Mi-RSNPs, could serve as valid instruments for the microbiota composition (exposure) in epidemiological studies and in formal one-sample or two-sample Mendelian randomization analyses [67]. Although other studies have examined the associations between several Mi-RNPs and different phenotypes (cardiovascular diseases, mental diseases, intestinal diseases, diabetes, etc.) using two-sample Mendelian randomization approaches [44,51,66,81,82,83,84,85,86], we used a different approach. In our study, instead of using summary data both for population 1 (testing the instrument–exposure association) and for population 2 (testing the instrument–outcome association), we undertook a new study in population 2 and were, therefore, able to obtain novel unexplored associations between Mi-RSNPs in a Mediterranean population (in addition to those reported in the UK Biobank or in other large consortia with publicly available summary data). This is important because it is known that genetic associations may differ depending on the geographical origin on the analyzed population [87], and more studies in diverse and understudied populations, such as the Mediterranean population, are needed. In addition, having the individual-level data for obtaining more results is an important advantage when performing genetic association studies because additional gene × diet or gene × sex interactions can be analyzed [67,68,70].

In our study, we focused on fasting plasma glucose and type 2 diabetes prevalence as outcomes to analyze the association between the host genetic variables previously reported to be related with gut microbiota taxa in four selected studies [43,45,46,51]. In this Mediterranean population, we observed some statistically significant associations at the nominal *p*-value between the selected Mi-RSNPs and fasting plasma glucose and type 2 diabetes. For fasting glucose, we analyzed the whole population, including nondiabetic subjects and type 2 diabetic subjects, adjusting for diabetes, medications, and other potential confounders such as sex, age, and BMI. In addition, we analyzed the associations only in nondiabetic subjects. For these analyses, we first examined the Mi-RSNPs as individual instruments, and no combined instruments, such as polygenic risk scores, were tested. The reason for this analysis is based on the existence of high heterogeneity in reporting the associations across the different publications and on the difficulties involved in deriving the effect allele and the direction of the effect across the studies [46]. Although very few Mi-RSNPs reached statistically significant associations with fasting plasma glucose or diabetes in our Mediterranean population, we detected some interesting associations. The hit associated with fasting glucose in the whole population was the Mi-RSNP rs9569095 located at a still uncharacterized gene (ncRNA gene), in chromosome 13, which has been related to the class *Gammaproteobacteria*_*HB* [45]. This class has been associated with diabetes in other studies directly analyzing the microbiome [88,89,90], but the results are not consistent. The other hit for fasting plasma glucose was an Mi-RSNP related to the order Rhizobiales [43]. This order is interesting because others Mi-RSNPs related with it [43] have reached statistical significance in determining fasting glucose in nondiabetic subjects and type 2 diabetes risk in this population. There are very few studies analyzing this order with diabetes-related traits [91,92]. This order, most abundant as free-living bacteria, is interesting as some studies have revealed an important role of this order in relevant pathways such as amino-acid metabolism, energy production and conversion, and carbohydrate metabolism [93]. Previous Mendelian randomization studies analyzing the association between Mi-RSNPs and type 2 diabetes have reported inconsistent results [51,66,84]. Yang et al. [66] analyzing the role of 27 genera of the human gut microbiota on type 2 diabetes and ischemic heart disease using Mendelian randomization, identified *Acidaminococcus*, *Aggregatibacter*, *Anaerostipes*, *Blautia*, *Desulfovibrio*, *Dorea*, and *Faecalibacterium* as being nominally associated with type 2 diabetes, whereas Xiang et al. [84] suggested that only Streptococcaceae was associated with higher type 2 diabetes risk in European populations after analyzing 28 gut microbiome families using a Mendelian randomization approach. This lack of consistency among the few studies analyzing the association between the Mi-RSNPs and type 2 diabetes may be due to the heterogeneity in the populations analyzed. We examined a high-cardiovascular-risk Mediterranean population, but some differences may exist when examining a healthy young population from the same Mediterranean area. In addition, we detected some sex × Mi-RSNP interactions, highlighting the need to better analyze the possible differences between men and women [94]. In addition, we observed some statistically significant Mi-RSNP × diet interactions in determining plasma fasting glucose when considering adherence to Mediterranean diet. Previous studies have reported the influence of diet on the effects of some Mi-RSNPs. This has been widely reported in the association between milk intake and the MCM6 Mi-RSNPs associated with different *Bifidobacterium* species and abundance [46,51]. Likewise, a high-fiber diet has been associated with the effect of ABO Mi-RSNPs on the abundance of *F. lactaris* [46]. More studies are needed to better analyze the dietary modulation of the Mi-RSNPs. Moreover, several studies directly analyzing microbiota composition and type 2 diabetes and related traits have been published, obtaining diverse results depending on the population studied [34,35,36,37,95]. Wang et al. [96], in a recent investigation performed in China, constructed a healthy microbiome index (HMI) and examined the relationship between the HMI and type 2 diabetes incidence. In our study, we also examined the effect of several genetic instruments on fasting glucose using a two-sample Mendelian randomization approach [67]. Selecting instrumental variables in the case of SNPs related to the microbiota is complex because the increase in the abundance of each microbial taxa can be beneficial or detrimental depending on the microorganism. As the effects of many of the taxa are still poorly understood, it is difficult to create combined instruments as genetic proxies. We analyzed a combination of eight Mi-RSNPs associated with significantly lower plasma fasting glucose in the Mediterranean population as the abundance of the microbial species represented by the Mi-RSNPs increases. Among them is the rs234545 Mi-RSNP, related with *Faecalibacterium prausnitzii* [51], a butyrate-producing bacteria that has been previously associated with lower type 2 diabetes risk. Additional studies focused not only on taxa SNPs but also on SNPs related to functionality are needed in different populations that also take into account sex, age, diet, and other lifestyle characteristics.

## 5. Conclusions

This work represents the first study carried out in a high-cardiovascular-risk Mediterranean population testing the association between Mi-RSNPs and plasma fasting glucose and type 2 diabetes. Our results identified a number of Mi-RSNPs significantly associated with fasting plasma glucose and type 2 diabetes contributing to the improvement of our understanding of the potential mechanisms driving these phenotypes. Moreover, we detected some Mi-RSNP interactions with adherence to the Mediterranean diet and with sex modulating the association between the Mi-RSNPs and fasting glucose, thereby eliciting new research hypotheses to be tested in future studies.

## Figures and Tables

**Table 1:** Demographic, clinical, and biochemical characteristics of the study population according to sex.

	Total (*n* = 1020)	Men (*n* = 371)	Women (*n* = 649)	*p*
Age (years)	66.9 ± 0.2	66.4 ± 0.3	67.2 ± 0.2	0.054
Weight (kg)	77.1 ± 0.4	81.8 ± 0.6	74.4 ± 0.5	<0.001
BMI (kg/m^2^)	30.6 ± 0.2	29.6 ± 0.2	31.2 ± 0.2	<0.001
SBP (mmHg)	147.2 ± 0.7	148.5 ± 1.1	146.4 ± 0.9	0.127
DBP (mmHg)	81.8 ± 0.3	82.5 ± 0.6	81.4 ± 0.4	0.106
Total cholesterol (mg/dL)	208.3 ± 1.2	200.5 ± 1.9	212.7 ± 1.6	<0.001
LDL-C (mg/dL)	129.5 ± 1.1	125.2 ± 1.8	131.9 ± 1.4	0.004
HDL-C (mg/dL)	52.7 ± 0.4	48.3 ± 0.6	55.2 ± 0.5	<0.001
Triglycerides ^1^ (mg/dL)	131.3 ± 2.2	135.1 ± 3.6	129.2 ± 2.7	0.191
Fasting glucose (mg/dL)	120.1 ± 1.2	126.4 ± 2.1	116.5 ± 1.5	<0.001
Total energy intake (kcal/day)	2203.6 ± 19.7	2336.9 ± 33.9	2127.3 ± 23.7	<0.001
Physical Activity (MET-min/day)	170.0 ± 5.4	228.6 ± 11.5	136.5 ± 5.0	<0.001
Adherence to MedDiet (P14) ^2^	8.5 ± 0.1	8.6 ± 0.1	8.4 ± 0.1	0.147
High Adherence MedDiet ^3^ (≥9)	505 (49.5)	187 (50.4)	318 (49.0)	0.666
Current smokers: *n*, %	128 (12.5)	99 (26.7)	29 (4.5)	<0.001
Type 2 diabetes: *n*, %	474 (46.5)	199 (53.6)	275 (42.4)	0.001

Values are the mean ± SE for continuous variables and number (%) for categorical variables. BMI: body mass index; SBP: systolic blood pressure; DBP: diastolic blood pressure; LDL-C: high-density lipoprotein cholesterol; HDL-C: low-density lipoprotein cholesterol; MET: metabolic equivalent (1 MET is equivalent to 1 kcal·kg^−1^·h^−1^, with the oxygen cost of sitting quietly measured as 3.5 mL/kg/min); *p*: *p*-value for the comparisons (means or percentages) between men and women. Student’s *t*-test was used to compare means, and chi-squared tests were used to compare categories. ^1^ Triglycerides were ln-transformed for statistical testing. ^2^ Quantitative 14-item questionnaire for adherence to Mediterranean diet. ^3^ High adherence represents a score ≥9 points on the P-14 scale.

**Table 2:** Association between the top-ranked microbiota-related SNPs and fasting glucose in the whole population ^1^.

Chr	SNP	Beta	*p*	MAF	Gene	Microbial Trait
13	rs9569095	−7.917	0.00099	0.144	LOC105370213	*C_Gammaproteobacteria_HB*
10	rs17103336	4.563	0.00593	0.219	BTBD16	*order_Rhizobiales*
4	rs11940694	−3.685	0.00918	0.499	KLB	*genus.Ruminococcustorquesgroup.id.14377*
14	rs10498633	4.845	0.01025	0.153	SLC24A4	*genus.Ruminococcustorquesgroup.id.14377*
1	rs10752747	3.258	0.03628	0.497	MMEL1	*genus.Tyzzerella3.id.11335*
17	rs2716882	3.737	0.05294	0.131	Intergenic	*genus_Sporacetigenium*
20	rs6108958	2.758	0.05605	0.359	LOC105372529	*genus_Abiotrophia*
20	rs6030140	−4.860	0.05747	0.139	PTPRT	*G_Holdemanella_HB*
2	rs1004787	2.674	0.06220	0.197	LOC107985879	*genus.Allisonella.id.2174*
4	rs2736990	−2.748	0.06539	0.393	SNCA	*phylum.Actinobacteria.id.400*
10	rs860912	3.173	0.07632	0.276	LOC105378531	*G_Subdoligranulum_HB*
1	rs867426	−2.812	0.07730	0.476	Intergenic	*G_unclassified_P_Proteobacteria_HB*
2	rs7580217	4.193	0.09186	0.242	KCNK12	*g__Parabacteroides*
9	rs1014306	−2.481	0.09196	0.184	DAPK1	*O_Rhodospirillales_RNT*
12	rs987019	−3.049	0.09574	0.193	PPM1H	*s__Romboutsia ilealis*
2	rs182549	−2.375	0.09772	0.163	MCM6	*s__Bifidobacterium adolescentis*
14	rs1951597	2.368	0.10150	0.480	LOC105370413	*G_Odoribacter_HB*
3	rs6766789	−3.874	0.10630	0.306	FHIT	*g__CAG-448*
6	rs3010562	2.291	0.12010	0.334	Intergenic	*genus_Anaerofilum*
6	rs2854275	−3.225	0.12090	0.065	HLA-DQB1	*genus.Streptococcus.id.1853*

^1^*N* = 1020 subjects. Model adjusted for sex, age, type 2 diabetes, BMI, and medication. Chr: chromosome. SNP: Single nucleotide polymorphism. Beta: the effect for the minor allele on fasting plasma glucose. *p*: *p*-value obtained in the multivariable linear regression model adjusted for sex, age, type 2 diabetes, BMI, and medication for each SNP using a genetic additive model. MAF: minor allele frequency in this population. BMI: Body mass index.

**Table 3:** Association between the top-ranked microbiota-related SNPs and type 2 diabetes in the whole population ^1^.

Chr	SNP	OR	*p*	MAF	Gene	Microbial Trait
10	rs17551124	0.775	0.00906	0.255	Intergenic	*order_Rhizobiales*
18	rs7235005	1.249	0.01406	0.443	LOC105372112	*G_unclassified_F_Erysipelotrichaceae_HB*
4	rs17063777	0.640	0.01939	0.162	Intergenic	*F_Desulfovibrionaceae_HB*
8	rs10090365	1.228	0.02043	0.403	Intergenic	*genus.CandidatusSoleaferrea.id.11350*
8	rs10091895	1.289	0.02241	0.428	CSMD1	*genus.Romboutsia.id.11347*
13	rs9569095	1.364	0.04048	0.144	LOC105370213	*C_Gammaproteobacteria_HB*
9	rs10780691	0.835	0.04640	0.345	NTRK2	*family.Oxalobacteraceae.id.2966*
4	rs11947461	1.200	0.04968	0.336	Intergenic	*o__Chloroflexales*
10	rs10994397	0.645	0.05116	0.160	ANK3	*order.Gastranaerophilales.id.1591*
5	rs10064431	1.191	0.05420	0.411	FAM172A	*genus.Romboutsia.id.11347*
20	rs1035177	0.818	0.05659	0.315	MACROD2	*g__Holdemania*
4	rs7654391	0.687	0.06058	0.096	Intergenic	*F_Desulfovibrionaceae_HB*
2	rs1507705	1.244	0.06471	0.380	DTNB	*genus_Desulfovibrio*
12	rs10777875	0.819	0.06689	0.472	RMST	*genus.Ruminococcus1.id.11373*
8	rs6468721	0.827	0.06831	0.152	Intergenic	*c__Syntrophorhabdia*
17	rs228770	0.691	0.07381	0.053	NAGS	*G_Parasutterella_HB*
20	rs6108958	1.176	0.07503	0.359	LOC105372529	*genus_Abiotrophia*

^1^*N* = 1020 subjects. Model adjusted for sex, age, and BMI. Chr: chromosome. SNP: Single nucleotide polymorphism. OR: odds ratio; ORs were calculated for the minor allele effect on type 2 diabetes risk. *p*: *p*-value obtained in the multi-variable logistic regression model adjusted for sex, age, and BMI for each SNP using a genetic additive model. MAF: minor allele frequency. BMI: Body mass index.

**Table 4:** Interaction between the top-ranked microbiota-related SNPs and Mediterranean diet on plasma fasting glucose in the whole population ^1^.

Chr	SNP	Beta 1	SE 1	Beta 2	SE 2	p_GenexDiet	MAF	Gene	Microbial Trait
17	rs2716882	12.440	3.485	−0.451	3.142	0.0060	0.1312	Intergenic	*genus_Sporacetigenium*
10	rs10823909	−4.936	3.900	7.916	3.563	0.0150	0.0379	ANAPC16	*g__CAG-776*
1	rs16833405	−5.816	3.839	5.812	3.202	0.0200	0.2568	Intergenic	*O_Rhodospirillales_HB*
2	rs7580217	7.420	4.211	−6.183	4.368	0.0250	0.2422	KCNK12	*g__Parabacteroides*
2	rs6717477	−3.776	2.605	3.563	2.299	0.0347	0.3391	Intergenic	*genus_Acinetobacter*
2	rs6546314	6.379	3.711	−4.390	3.577	0.0367	0.2274	DTNB	*genus_Desulfovibrio*
11	rs7104796	−6.851	8.103	15.920	7.374	0.0376	0.0986	LDLRAD3	*F_Sutterellaceae_HB*
4	rs10520163	3.063	2.722	−4.335	2.337	0.0392	0.4655	CLCN3	*genus.Allisonella.id.2174*
6	rs7773795	−10.310	4.327	1.553	3.979	0.0436	0.0962	RTN4IP1	*g__Achromobacter*
9	rs11141878	4.587	2.693	−2.560	2.419	0.0483	0.4209	DAPK1	*O_Rhodospirillales_RNT*
16	rs12597384	13.820	6.666	−2.754	5.129	0.0488	0.0519	Intergenic	*G_Odoribacter_HB*
13	rs9569095	1.127	4.270	−10.350	4.022	0.0504	0.1442	LOC105370213	*C_Gammaproteobacteria_HB*

^1^*N* = 1020 subjects. Chr: chromosome. SNP: Single nucleotide polymorphism. Beta: effect for the minor allele on fasting plasma glucose concentrations SE: Standard error. Beta 1 and SE 1 indicate the regression coefficients and SE for the low adherence to Mediterranean diet strata (50%). Beta 2 and SE 2 indicate the regression coefficients for the high adherence to Mediterranean diet strata, based on the population mean (9 points). p_GenexDiet: indicates the *p*-value for the interaction term between each SNP and adherence to the Mediterranean diet in the corresponding hierarchical general linear regression model including the main effects and interaction terms in the whole population. MAF: minor allele frequency.

**Table 5:** Mendelian randomization estimates for each method of the so-called “causal” effect of gut microbiome (nine selected instruments on fasting plasma glucose).

Method	Estimate	SE	95%_CI		*p*-Value
Simple_median	−35.841	19.400	−73.863	2.182	0.065
Weighted_median	−25.292	15.332	−55.342	4.759	0.099
Penalized_weighted_median	−29.185	15.782	−60.117	1.747	0.064
IVW	−17.198	14.415	−45.451	11.055	0.233
Penalized_IVW	−25.517	12.300	−49.626	−1.409	0.038
Robust_IVW	−19.300	13.537	−45.832	7.232	0.154
Penalized_robust_IVW	−25.811	8.198	−41.879	−9.743	0.002
MR-Egger	−21.692	26.192	−73.027	29.644	0.408
(intercept)	0.351	1.657	−2.896	3.598	0.832
Penalized_MR-Egger	−21.692	26.192	−73.027	29.644	0.408
(intercept)	0.351	1.657	−2.896	3.598	0.832
Robust_MR-Egger	−23.403	18.326	−59.321	12.515	0.202
(intercept)	0.363	1.287	−2.159	2.885	0.778
Penalized_robust_MR-Egger	−23.403	18.326	−59.321	12.515	0.202
(intercept)	0.363	1.287	−2.159	2.885	0.778

CI: confidence interval. IVW: inverse variance weighted method. SE: Standard error. MR: Mendelian randomization.

## Data Availability

Neither the participants’ consent forms nor ethics approval included permission for open access. However, we followed a controlled data-sharing collaboration model, and data for collaborations will be available upon request pending application and approval. Investigators who are interested in this study can contact the last author Dolores Corella (dolores.corella@uv.es).

## Supplementary Materials

The following supporting information can be downloaded at https://www.mdpi.com/article/10.3390/medicina58091238/s1: Table S1. Microbiome-related SNPs selected for this study: initial mbGWAS generating the association (STD#), SNP location, and microbial trait; Table S2. Association between the ordered (by *p*-value) microbiota-related SNPs and fasting glucose in the whole population1 (continuation of Table 2); Table S3. Association between the microbiota-related SNPs and diabetes in nondiabetic subjects; Table S4. Interaction between the top-ranked microbiota-related SNPs and sex on plasma fasting glucose in the whole population; Table S5. Selected instruments for the two-sample Mendelian randomization analysis on plasma glucose.
