# Supplementary material for: Association between Microbiome-Related Human Genetic Variants and Fasting Plasma Glucose in a High-Cardiovascular-Risk Mediterranean Population
Source: Medicina (Kaunas). 2022 Sep 7;58(9):1238. doi: 10.3390/medicina58091238 (PMC9502852; doi:10.3390/medicina58091238)
Supplement: Supplementary file 1 [file medicina-58-01238-s001.zip › medicina-1875762-supplementary.pdf]

Article

Association between microbiome-related human genetic variants and fasting plasma glucose in a high-cardiovascular risk Mediterranean population

Eva M Asensio, Carolina Ortega-Azorín, Rocío Barragán, Andrea Alvarez-Sala, José V. Sorlí, Eva C. Pascual, Rebeca Fernández-Carrión, Laura V Villamil, Dolores Corella, Oscar Coltell

SUPPLEMENTAL TABLES AND FIGURES

Table S1. Microbiome-related SNPs selected for this study: initial mbGWAS generating the association (STD#), SNP location and microbial trait.

Table S2. Association between the ordered (by p-value) microbiota-related SNPs and fasting glucose in the whole population1 (continuation of Table 2)1.

Table S3. Association between the microbiota-related SNPs and diabetes in non-diabetic subjects1.

Table S4. Interaction between the top-ranked microbiota-related SNPs and sex on plasma fasting glucose in the whole population1.

Table S5. Selected instruments for the Two-sample Mendelian randomization analysis on plasma glucose.

Table S1. Microbiome-related SNPs selected for this study: initial mbGWAS generating the association (STD#), SNP location and microbial trait.

Study code	Microbial trait	SNP	Chr	Gene
STD2	*genus.Allisonella.id.2174*	rs1004787	2	LOC107985879
STD2	*genus.Romboutsia.id.11347*	rs10064431	5	FAM172A
STD2	*genus.Allisonella.id.2174*	rs10087900	8	intergenic
STD2	*genus.CandidatusSoleaferrea.id.11350*	rs10090365	8	intergenic
STD2	*genus.Romboutsia.id.11347*	rs10091895	8	CSMD1
STD2	*genus.Faecalibacterium.id.2057*	rs10127720	1	intergenic
STD2	*genus..Ruminococcustorquesgroup.id.14377*	rs1014174	2	PID1
STD1	*O_Rhodospirillales_RNT*	rs1014306	9	DAPK1
STD2	*genus.Ruminococcus1.id.11373*	rs1014486	3	IL12A-AS1
STD4	*genus_Desulfovibrio*	rs10173922	2	LOC105373456
STD2	*genus.Erysipelatoclostridium.id.11381*	rs10185424	2	intergenic
STD4	*family_Pasteurellaceae*	rs1021993	1	intergenic
STD2	*genus..Eubacteriumcoprostanoligenesgroup.id.11375*	rs10241138	7	ADCYAP1R1
STD2	*genus.Peptococcus.id.2037*	rs1024582	12	CACNA1C
STD2	*class.Actinobacteria.id.419*	rs10267710	7	intergenic
STD2	*genus.Streptococcus.id.1853*	rs10273733	7	BCAP29
STD2	*genus.Romboutsia.id.11347*	rs10279978	7	SLC29A4
STD3	*g__Holdemania*	rs1035177	20	MACROD2
STD2	*genus..Ruminococcustorquesgroup.id.14377*	rs10401969	19	SUGP1
STD2	*genus.Erysipelatoclostridium.id.11381*	rs1042058	10	MAP3K8
STD2	*genus..Eubacteriumcoprostanoligenesgroup.id.11375*	rs10444197	10	LOC105376348
STD2	*genus.Faecalibacterium.id.2057*	rs10488631	7	TNPO3
STD4	*genus_Fusobacterium*	rs10489747	1	RXRG
STD4	*genus_Eubacterium*	rs10490411	2	intergenic
STD4	*family_Pasteurellaceae*	rs10494924	1	intergenic
STD4	*genus_Coprococcus*	rs10496481	2	DPP10
STD4	*genus_Fusobacterium*	rs10498546	14	CEP128
STD2	*genus..Ruminococcustorquesgroup.id.14377*	rs10498633	14	SLC24A4
STD4	*order_Selenomonadales*	rs10516879	4	CCSER1
STD2	*genus.Allisonella.id.2174*	rs10520163	4	CLCN3
STD2	*order.Gastranaerophilales.id.1591*	rs1062633	3	MST1R
STD2	*genus.Oxalobacter.id.2978*	rs10739743	9	intergenic
STD2	*genus.Tyzzerella3.id.11335*	rs10752747	1	MMEL1
STD2	*genus.Ruminococcus1.id.11373*	rs10777875	12	RMST
STD2	*family.Oxalobacteraceae.id.2966*	rs10780691	9	NTRK2
STD2	*genus.Intestinibacter.id.11345*	rs10805326	4	intergenic
STD2	*genus.Allisonella.id.2174*	rs10828248	10	MLLT10
STD2	*order.Bifidobacteriales.id.432*	rs10831953	11	intergenic
STD2	*genus.Oxalobacter.id.2978*	rs10868238	9	NTRK2
STD2	*genus.Erysipelatoclostridium.id.11381*	rs10868677	9	intergenic
STD2	*class.Actinobacteria.id.419*	rs10871420	16	LOC105369213
STD3	*g__Hydrogenophaga*	rs10914206	1	intergenic
STD2	*order.Gastranaerophilales.id.1591*	rs10994397	10	ANK3
STD4	*genus_Coprococcus*	rs11123259	2	DPP10
STD4	*genus_Lactobacillus*	rs11126954	2	LOC107985905
STD1	*O_Rhodospirillales_RNT*	rs11141878	9	DAPK1

Table S1. Microbiome-related SNPs selected for this study: initial mbGWAS generating the association (STD#), SNP location and microbial trait.

Study code	Microbial trait	SNP	Chr	Gene
STD1	*G_Faecalitalea_HB*	rs11164526	1	intergenic
STD3	*g__Gillisia*	rs11165262	1	TGFBR3
STD4	*order_Selenomonadales*	rs11203893	8	MTUS1
STD1	*G_Dialister_HB*	rs11218350	11	SORL1
STD2	*family.Oxalobacteraceae.id.2966*	rs11246212	11	PHRF1
STD4	*genus_Anaerotruncus*	rs11696277	20	PCSK2
STD1	*G_Clostridium_sensu_stricto_HB*	rs11865270	16	ZC3H18
STD4	*order_Selenomonadales*	rs11935423	4	TRMT10A
STD2	*genus..Ruminococcustorquesgroup.id.14377*	rs11940694	4	KLB
STD3	*o__Chloroflexales*	rs11947461	4	intergenic
STD1	*G_Aestuariispira_HB*	rs11980485	7	PDE1C
STD1	*O_Rhodospirillales_RNT*	rs12001404	9	DAPK1
STD1	*C_Deltaproteobacteria_HB*	rs12004427	9	LOC105376214
STD1	*G_Ruminococcus_HB*	rs12051754	17	MIR4521
STD3	*s__Megasphaera elsdenii*	rs12076104	1	CASZ1
STD4	*family_Carnobacteriaceae*	rs12145418	1	ESRRG
STD2	*order.Bifidobacteriales.id.432*	rs12188164	5	AHRR
STD2	*genus.RuminococcaceaeUCG013.id.11370*	rs12336782	9	intergenic
STD4	*genus_Phascolarctobacterium*	rs12367572	12	NELL2
STD1	*O_Rhodospirillales_RNT*	rs12378686	9	DAPK1
STD1	*F_Lachnospiraceae_RNT*	rs12490796	3	LOC105374165
STD2	*genus.RuminococcaceaeUCG009.id.11366*	rs12508214	4	SORCS2
STD4	*family_Enterobacteriaceae*	rs12530266	6	NKAIN2
STD1	*G_Odoribacter_HB*	rs12597384	16	intergenic
STD3	*g__Parabacteroides*	rs12673506	7	CHN2
STD2	*genus.Tyzzerella3.id.11335*	rs12713341	2	LOC105377632
STD4	*genus_Lactobacillus*	rs12714120	2	LOC107985905
STD2	*genus.CandidatusSoleaferrea.id.11350*	rs1295686	5	IL13
STD3	*g__UBA2821*	rs12995607	2	intergenic
STD3	*g__Herbidospora*	rs13097088	3	intergenic
STD4	*order_Selenomonadales*	rs13132148	4	intergenic
STD1	*G_Fusicatenibacter_RNT*	rs13136928	4	LRBA
STD1	*F_Enterococcaceae_HB*	rs1318969	11	KIRREL3
STD1	*G_unclassified_P_Firmicutes_RNT*	rs1333344	9	CTNNAL1
STD1	*O_Rhodospirillales_HB*	rs13375139	1	intergenic
STD4	*genus_Sporacetigenium*	rs13423976	2	intergenic
STD3	*f__Bin127*	rs134974	22	intergenic
STD2	*genus.Peptococcus.id.2037*	rs1421085	16	FTO
STD4	*family_Enterobacteriaceae*	rs1490359	6	NKAIN2
STD4	*genus_Desulfovibrio*	rs1507705	2	DTNB
STD2	*genus..Ruminococcustorquesgroup.id.14377*	rs158487	5	intergenic
STD1	*O_Rhodospirillales_HB*	rs16833405	1	intergenic
STD1	*F_Sutterellaceae_HB*	rs16844464	4	DOK7
STD1	*O_Rhodospirillales_RNT*	rs16877042	4	ZCCHC4
STD1	*G_Dialister_HB*	rs1699103	11	SORL1
STD4	*genus_Eubacterium*	rs17039629	2	intergenic

Table S1. Microbiome-related SNPs selected for this study: initial mbGWAS generating the association (STD#), SNP location and microbial trait.

Study code	Microbial trait	SNP	Chr	Gene
STD1	*F_Desulfovibrionaceae_HB*	rs17063777	4	intergenic
STD1	*F_Sutterellaceae_HB*	rs17068792	5	TENM2
STD1	*G_Holdemanella_HB*	rs17081294	6	LOC107986525
STD4	*order_Rhizobiales*	rs17103336	10	BTBD16
STD1	*G_Ruminococcus_HB*	rs17121264	11	FXYD6-FXYD2
STD4	*genus_Desulfovibrio*	rs17259859	10	intergenic
STD4	*genus_Olsenella*	rs17505775	3	TP63
STD2	*genus.Oxalobacter.id.2978*	rs17543178	6	BTBD9
STD4	*order_Rhizobiales*	rs17551124	10	intergenic
STD2	*genus.Tyzzerella3.id.11335*	rs17706248	5	LOC101929505
STD1	*G_Dialister_HB*	rs1790213	11	SORL1
STD1	*G_Dialister_HB*	rs1792113	11	SORL1
STD3	*s__Bifidobacterium adolescentis*	rs182549	2	MCM6
STD4	*order_Selenomonadales*	rs1922233	4	CCSER1
STD1	*G_Odoribacter_HB*	rs1951597	14	LOC105370413
STD1	*G_Dialister_HB*	rs2070045	11	SORL1
STD2	*genus..Ruminococcustorquesgroup.id.14377*	rs2109479	5	intergenic
STD3	*g__Prevotellamassilia*	rs2140551	2	STON1-GTF2A1L
STD3	*s__Eubacterium_I ramulus_A*	rs2194316	16	LINC01572
STD4	*genus_Holdemania*	rs2271001	11	ZDHHC13
STD1	*G_unclassified_P_Firmicutes_RNT*	rs2271878	9	TMEM245
STD3	*c__Paceibacteria*	rs2277424	13	LINC00346
STD1	*G_Parasutterella_HB*	rs228752	17	LSM12
STD1	*G_Parasutterella_HB*	rs228770	17	NAGS
STD3	*s__Faecalibacterium prausnitzii_E*	rs234545	15	intergenic
STD4	*genus_Phascolarctobacterium*	rs2541522	16	TEKT5
STD4	*family_Succinivibrionaceae*	rs2584	8	MCPH1
STD1	*G_unclassified_C_Clostridia_RNT*	rs267786	5	intergenic
STD4	*genus_Sporacetigenium*	rs2716882	17	intergenic
STD2	*phylum.Actinobacteria.id.400*	rs2736990	4	SNCA
STD1	*G_Butyricicoccus_RNT*	rs28540434	16	ARHGAP17
STD2	*genus.Streptococcus.id.1853*	rs2854275	6	HLA-DQB1
STD1	*G_Bifidobacterium_HB*	rs2855306	3	THPO
STD1	*G_Clostridium_sensu_stricto_RNT*	rs2858332	6	intergenic
STD1	*O_Rhodospirillales_RNT*	rs2875425	4	ZCCHC4
STD4	*genus_Lachnobacterium*	rs2921739	8	intergenic
STD1	*F_Sutterellaceae_HB*	rs2938462	5	intergenic
STD4	*genus_Anaerofilum*	rs3010562	6	intergenic
STD3	*s__UBA2922 sp900313925*	rs35262813	16	intergenic
STD2	*order.Gastranaerophilales.id.1591*	rs367480	11	SLC22A18
STD4	*genus_Coprococcus*	rs3739034	2	intergenic
STD4	*genus_Lactococcus*	rs3747113	22	SPECC1L
STD1	*G_unclassified_P_Firmicutes_RNT*	rs3818932	9	IKBKAP
STD1	*G_Aestuariispira_RNT*	rs3852540	12	LOC105369710
STD1	*G_Streptococcus_HB*	rs4300218	1	PIK3CD
STD1	*G_Dialister_HB*	rs4420280	11	SORL1

Table S1. Microbiome-related SNPs selected for this study: initial mbGWAS generating the association (STD#), SNP location and microbial trait.

Study code	Microbial trait	SNP	Chr	Gene
STD1	*G_Fusicatenibacter_RNT*	rs4458434	4	LRBA
STD4	*genus_Bifidobacterium*	rs4479964	6	ARHGAP18
STD4	*genus_Desulfovibrio*	rs4614937	2	LOC105373456
STD4	*genus_Acinetobacter*	rs4662863	2	intergenic
STD4	*order_Rhizobiales*	rs4665809	2	intergenic
STD4	*order_Selenomonadales*	rs4705	8	PDGFRL
STD1	*F_Sutterellaceae_HB*	rs4756282	11	LDLRAD3
STD1	*G_Butyricicoccus_RNT*	rs4788439	16	ARHGAP17
STD1	*G_Streptococcus_HB*	rs4799294	18	intergenic
STD4	*genus_Akkermansia*	rs4894707	3	PLD1
STD3	*s__Rubneribacter sp002159915*	rs4925523	1	intergenic
STD1	*G_Dialister_HB*	rs4936637	11	SORL1
STD3	*s__Bifidobacterium bifidum*	rs4988235	2	MCM6
STD4	*genus_Desulfovibrio*	rs517403	2	DTNB
STD1	*G_unclassified_P_Bacteroidetes_HB*	rs561060	6	MTHFD1L
STD1	*G_Desulfovibrio_HB*	rs583852	11	intergenic
STD1	*G_Holdemanella_HB*	rs6030140	20	PTPRT
STD4	*genus_Abiotrophia*	rs6108958	20	LOC105372529
STD4	*genus_Anaerotruncus*	rs6111497	20	PCSK2
STD1	*G_Dialister_HB*	rs639648	11	SORL1
STD1	*G_Parasutterella_HB*	rs640631	17	NAGS
STD1	*G_Senegalimassilia_HB*	rs6441647	3	intergenic
STD4	*genus_Desulfovibrio*	rs6463094	7	intergenic
STD3	*c__Syntrophorhabdia*	rs6468721	8	intergenic
STD4	*genus_Desulfovibrio*	rs6531212	2	intergenic
STD1	*G_unclassified_F_Erysipelotrichaceae_HB*	rs6545561	2	CCDC85A
STD4	*genus_Desulfovibrio*	rs6546314	2	DTNB
STD1	*G_Clostridium_sensu_stricto_RNT*	rs6562211	13	RFC3
STD1	*C_Actinobacteria_RNT*	rs6570118	6	intergenic
STD4	*genus_Bifidobacterium*	rs6683441	1	intergenic
STD4	*genus_Acinetobacter*	rs6717477	2	intergenic
STD1	*C_Alphaproteobacteria_RNT*	rs6723629	2	CD207
STD1	*G_Bifidobacterium_RNT*	rs6730157	2	RAB3GAP1
STD1	*G_unclassified_F_Erysipelotrichaceae_HB*	rs6733298	2	CCDC85A
STD4	*genus_Desulfovibrio*	rs6746082	2	DTNB
STD4	*genus_Coprococcus*	rs6747870	2	TMEM163
STD3	*g__CAG-448*	rs6766789	3	FHIT
STD3	*s__Anaeromassilibacillus sp001305115*	rs6797860	3	TP63
STD3	*s__Massiliomicrobiota sp002160815*	rs6890044	5	intergenic
STD1	*G_Blautia_RNT*	rs6891143	5	ARAP3
STD4	*genus_Bifidobacterium*	rs6999452	8	ZFPM2
STD1	*F_Sutterellaceae_HB*	rs7104796	11	LDLRAD3
STD1	*G_Blautia_RNT*	rs7129903	11	intergenic
STD1	*G_unclassified_F_Erysipelotrichaceae_HB*	rs7235005	18	LOC105372112
STD1	*G_unclassified_K_Bacteria_RNT*	rs7245009	18	DOK6
STD1	*C_Alphaproteobacteria_RNT*	rs7565578	2	CD207

Table S1. Microbiome-related SNPs selected for this study: initial mbGWAS generating the association (STD#), SNP location and microbial trait.

Study code	Microbial trait	SNP	Chr	Gene
STD1	*F_Sutterellaceae_HB*	rs7568213	2	intergenic
STD3	*g__Parabacteroides*	rs7580217	2	KCNK12
STD3	*s__Lawsonibacter sp002161175*	rs7631252	3	intergenic
STD1	*F_Desulfovibrionaceae_HB*	rs7654391	4	intergenic
STD1	*G_Fusicatenibacter_RNT*	rs7657948	4	LRBA
STD1	*G_Holdemanella_HB*	rs7740164	6	LOC107986525
STD3	*g__Achromobacter*	rs7773795	6	RTN4IP1
STD3	*g__CAG-632*	rs7958254	12	intergenic
STD3	*f__TMED109*	rs7973111	12	LOC105369901
STD4	*order_Rhizobiales*	rs8037033	15	FMN1
STD4	*genus_Bifidobacterium*	rs8056064	16	CDH13
STD4	*genus_Bifidobacterium*	rs8063330	16	intergenic
STD1	*F_Desulfovibrionaceae_HB*	rs820136	17	MYO15B
STD1	*G_Subdoligranulum_HB*	rs860912	10	LOC105378531
STD1	*G_unclassified_P_Proteobacteria_HB*	rs867426	1	intergenic
STD4	*genus_Faecalibacterium*	rs910633	1	intergenic
STD4	*genus_Desulfovibrio*	rs936012	2	DTNB
STD4	*genus_Lachnobacterium*	rs9363741	6	LOC105377842
STD4	*family_Enterobacteriaceae*	rs9401713	6	NKAIN2
STD3	*f__Geobacteraceae*	rs9482647	6	LOC107986640
STD1	*G_Clostridium_sensu_stricto_RNT*	rs9563941	13	RFC3
STD1	*C_Gammaproteobacteria_HB*	rs9569095	13	LOC105370213
STD1	*G_Clostridium_sensu_stricto_RNT*	rs9570673	13	LOC105370156
STD3	*s__Romboutsia ilealis*	rs987019	12	PPM1H
STD4	*genus_Desulfovibrio*	rs9886211	7	GLI3

Chr: Chromosome.

STD1: Hughes DA, et al. 2020. [45]

STD2: Kurilshikov A, et al. 2021. [46]

STD3: Qin Y, et al. 2022. [51]

STD4: Davenport ER, et al. 2015. [43]

Table S2. Association between the ordered (by p-value) microbiota-related SNPs and fasting glucose in the whole population1 (continuation of Table 2)[1].

Chr	SNP	Beta	*p*	MAF	Gene	Microbial trait
5	rs2938462	2.170	0.12850	0.281	*intergenic*	*F_Sutterellaceae_HB*
16	rs1186527(	-2.700	0.13160	0.179	ZC3H18	*G_Clostridium_sensu_stricto_HB*
5	rs6890044	3.488	0.14030	0.082	*intergenic*	*s__Massiliomicrobiota sp002160815*
1	rs1116452(	3.592	0.14140	0.159	*intergenic*	*G_Faecalitalea_HB*
2	rs4988235	-2.098	0.14490	0.161	MCM6	*s__Bifidobacterium bifidum*
2	rs6746082	-2.722	0.15160	0.385	DTNB	*genus_Desulfovibrio*
2	rs517403	-2.493	0.15490	0.328	DTNB	*genus_Desulfovibrio*
2	rs1271334:	3.150	0.16060	0.209	LOC105377632	*genus.Tyzzerella3.id.11335*
6	rs7773795	-3.374	0.16170	0.096	RTN4IP1	*g__Achromobacter*
7	rs9886211	2.170	0.16510	0.228	GLI3	*genus_Desulfovibrio*
8	rs2921739	1.903	0.19660	0.494	*intergenic*	*genus_Lachnobacterium*
10	rs1755112[illegible]	1.988	0.19680	0.255	*intergenic*	*order_Rhizobiales*
4	rs1687704:	3.382	0.20970	0.090	ZCCHC4	*O_Rhodospirillales_RNT*
2	rs1014174	1.726	0.21380	0.301	PID1	*genus..Ruminococcustorquesgroup.id.14377*
15	rs234545	1.912	0.21410	0.349	*intergenic*	*s__Faecalibacterium prausnitzii_E*
3	rs1014486	1.804	0.21800	0.321	IL12A-AS1	*genus.Ruminococcus1.id.11373*
5	rs2109479	-2.215	0.22410	0.277	*intergenic*	*genus..Ruminococcustorquesgroup.id.14377*
2	rs936012	-2.143	0.23160	0.370	DTNB	*genus_Desulfovibrio*
1	rs1116526:	2.029	0.24240	0.126	TGFBR3	*g__Gillisia*
6	rs9401713	-1.792	0.24590	0.391	NKAIN2	*family_Enterobacteriaceae*
2	rs4662863	-2.005	0.24940	0.283	*intergenic*	*genus_Acinetobacter*
1	rs1048974[illegible]	-3.714	0.25110	0.075	RXRG	*genus_Fusobacterium*
18	rs7245009	-2.057	0.25180	0.218	DOK6	*G_unclassified_K_Bacteria_RNT*
11	rs4756282	5.759	0.25480	0.066	LDLRAD3	*F_Sutterellaceae_HB*
2	rs1299560[illegible]	2.763	0.25590	0.063	*intergenic*	*g__UBA2821*
8	rs1009189[illegible]	-1.967	0.26530	0.428	CSMD1	*genus.Romboutsia.id.11347*
2	rs6730157	-1.598	0.26540	0.158	RAB3GAP1	*G_Bifidobacterium_RNT*
2	rs1017392:	-1.624	0.26920	0.493	LOC105373456	*genus_Desulfovibrio*
1	rs1214541[illegible]	1.668	0.27180	0.262	ESRRG	*family_Carnobacteriaceae*
9	rs1200140[illegible]	-1.706	0.27480	0.151	DAPK1	*O_Rhodospirillales_RNT*
8	rs2584	-1.667	0.28280	0.132	MCPH1	*family_Succinivibrionaceae*
6	rs1253026(	-1.631	0.28470	0.456	NKAIN2	*family_Enterobacteriaceae*
4	rs2875425	2.694	0.29050	0.161	ZCCHC4	*O_Rhodospirillales_RNT*
4	rs7654391	3.220	0.29580	0.096	*intergenic*	*F_Desulfovibrionaceae_HB*
7	rs1026771(	-1.551	0.30020	0.432	*intergenic*	*class.Actinobacteria.id.419*
4	rs1706377[illegible]	2.962	0.30470	0.162	*intergenic*	*F_Desulfovibrionaceae_HB*
18	rs4799294	2.772	0.30610	0.307	*intergenic*	*G_Streptococcus_HB*
17	rs820136	3.710	0.30870	0.123	MYO15B	*F_Desulfovibrionaceae_HB*
9	rs1237868(	-1.556	0.31340	0.142	DAPK1	*O_Rhodospirillales_RNT*
11	rs1790213	-1.453	0.32120	0.336	SORL1	*G_Dialister_HB*
4	rs1684446[illegible]	-3.617	0.33380	0.081	DOK7	*F_Sutterellaceae_HB*
11	rs1121835(	-1.696	0.33500	0.339	SORL1	*G_Dialister_HB*

[1]: N = 1020 subjects. Model adjusted for sex, age, type 2 diabetes, BMI and medication. Chr: Chromosome. Beta: indicates the effect for the minor allele on fasting plasma glucose. p: P-value obtained in the multi-variable linear regression model adjusted for sex, age, type 2 diabetes, BMI and medication for each SNP using a genetic additive model. MAF: Minor Allele Frequency in this population.

Note: this table is the continuation of Table 2, then only SNPs from p>0.1209 to p≤0.3350 are shown.

Table S3. Association between the microbiota-related SNPs and diabetes in non-diabetic subjects [1].

Chr	SNP	Beta	*p*	MAF	Gene	Microbial trait
5	rs6890044	5.464	0.00317	0.082	__	s__Massiliomicrobiota sp002160815
12	rs987019	-2.840	0.04434	0.193	PPM1H	s__Romboutsia ilealis
1	rs1091420[illegible]	-1.989	0.09038	0.349	__	g__Hydrogenophaga
16	rs3526281[illegible]	2.637	0.11750	0.070	__	s__UBA2922 sp900313925
2	rs182549	-1.512	0.17800	0.163	MCM6	s__Bifidobacterium adolescentis
6	rs7773795	-2.309	0.21490	0.096	RTN4IP1	g__Achromobacter
2	rs4988235	-1.393	0.21570	0.161	MCM6	s__Bifidobacterium bifidum
12	rs7973111	2.601	0.22580	0.104	LOC105369901	f__TMED109
15	rs234545	1.472	0.23090	0.349	__	s__Faecalibacterium prausnitzii_E
2	rs7580217	2.353	0.24010	0.242	KCNK12	g__Parabacteroides
8	rs6468721	1.349	0.27460	0.152	__	c__Syntrophorhabdia
3	rs6797860	-1.273	0.31740	0.360	TP63	s__Anaeromassilibacillus sp001305115
1	rs1116526[illegible]	1.336	0.31990	0.126	TGFBR3	g__Gillisia
3	rs6766789	-1.665	0.35300	0.306	FHIT	g__CAG-448
2	rs2140551	-0.926	0.41450	0.486	STON1-GTF2A1L	g__Prevotellamassilia
22	rs134974	-0.901	0.42630	0.436	__	f__Bin127
1	rs4925523	-0.983	0.44050	0.430	__	s__Rubneribacter sp002159915
6	rs9482647	-0.991	0.47780	0.195	LOC107986640	f__Geobacteraceae
13	rs2277424	-1.050	0.52620	0.191	LINC00346	c__Paceibacteria
3	rs7631252	0.729	0.64950	0.215	__	s__Lawsonibacter sp002161175
12	rs7958254	1.415	0.67990	0.135	__	g__CAG-632
7	rs1267350[illegible]	-0.426	0.74820	0.243	CHN2	g__Parabacteroides
2	rs1299560[illegible]	0.617	0.75150	0.063	__	g__UBA2821
20	rs1035177	0.384	0.76690	0.315	MACROD2	g__Holdemania
1	rs1207610[illegible]	-1.079	0.77280	0.066	CASZ1	s__Megasphaera elsdenii

[1]: N = 546 subjects. Model adjusted for sex, age and BMI. Chr: Chromosome. Beta: indicates the effect for the minor allele on fasting plasma glucose. *p* : P-value obtained in the multi-variable linear regression model adjusted for sex, age and BMI for each SNP using a genetic additive model. MAF: Minor Allele Frequency in this population.

Table S4. Interaction between the top-ranked microbiota-related SNPs and sex on plasma fasting glucose in the whole population[1].

Chr	SNP	Beta1	SE1	Beta2	SE2	*p* _GxSex	MAF	Gene	Microbial trait
11	rs367480	7.795	2.959	-3.502	2.112	0.0019	0.438	SLC22A18	order.Gastranaerophilales.id.1591
11	rs7129903	13.170	5.414	-4.182	3.627	0.0078	0.248	*intergenic*	G_Blautia_RNT
4	rs1313214[illegible]	-9.893	4.013	3.131	2.865	0.0083	0.157	*intergenic*	order_Selenomonadales
16	rs1186527[illegible]	2.527	3.612	-8.265	2.725	0.0171	0.179	ZC3H18	G_Clostridium_sensu_stricto_HB
6	rs9401713	-8.307	3.212	1.058	2.299	0.0178	0.391	NKAIN2	family_Enterobacteriaceae
6	rs1253026[illegible]	-7.272	3.173	1.843	2.271	0.0195	0.456	NKAIN2	family_Enterobacteriaceae
6	rs1490359	-6.810	3.141	2.075	2.274	0.0220	0.448	NKAIN2	family_Enterobacteriaceae
6	rs4479964	-5.463	2.926	2.681	2.082	0.0233	0.344	ARHGAP18	genus_Bifidobacterium
4	rs1194069[illegible]	-9.675	2.916	-1.583	2.120	0.0248	0.499	KLB	genus..Ruminococcustorquesgroup.id.14377
6	rs853964	11.090	6.714	-9.684	6.667	0.0281	0.010	LOC105377992	C_Deltaproteobacteria_HB
11	rs1699103	5.478	3.071	-2.588	2.182	0.0323	0.267	SORL1	G_Dialister_HB
16	rs1259738[illegible]	20.270	8.405	-0.272	4.666	0.0327	0.052	*intergenic*	G_Odoribacter_HB
11	rs639648	5.173	3.091	-2.687	2.177	0.0376	0.260	SORL1	G_Dialister_HB
11	rs4756282	28.990	11.970	1.272	7.091	0.0464	0.066	LDLRAD3	F_Sutterellaceae_HB
15	rs234545	-2.303	3.388	5.542	2.222	0.0529	0.349	*intergenic*	s__Faecalibacterium prausnitzii_E

[1]: N = 1020 subjects. Chr: Chromosome. Beta: indicates the effect for the minor allele on fasting plasma glucose concentrations. Beta 1 and SE 1: indicates the regression coefficients and SE in men (50%). Beta 2 and SE 2: indicates the regression coefficients and SE in women. p_GxSex: indicates the p-value for the interaction term between each SNP and sex in the corresponding hierarchical general linear regression model including the main effects and interaction terms in the whole population. MAF: minor allele frequency.

Table S5. Selected instruments for the Two-sample Mendelian randomization analysis on plasma glucose.

		Exposure (Microbiome)[1]					Outcome (Fasting glucose)[2]			
**SNP**	**Microbial trait**	**Beta**	**SE**	**EF**	**MAF**	***p***	**Beta**	**SE**	**MAF**	***p***
rs10914206	g__Hydrogenophaga	0.027	0.005	T	0.284	1.70E-08	1.446	1.828	0.349	0.4291
rs11947461	o__Chloroflexales	0.021	0.004	T	0.393	2.90E-08	1.833	1.804	0.336	0.3097
rs12673506	g__Parabacteroides	0.102	0.018	A	0.201	8.00E-09	-1.493	2.071	0.243	0.4712
rs234545	s__Faecalibacterium prausnitzii_E	0.051	0.009	C	0.445	2.40E-08	-2.325	1.874	0.349	0.2150
rs6468721	c__Syntrophorhabdia	0.024	0.004	C	0.265	3.50E-08	-2.662	2.005	0.152	0.1846
rs6766789	g__CAG-448	0.158	0.027	A	0.083	9.10E-09	-5.660	2.933	0.306	0.0539
rs7773795	g__Achromobacter	0.034	0.006	C	0.095	4.00E-08	-4.571	2.951	0.096	0.1217
rs9482647	f__Geobacteraceae	0.035	0.006	G	0.098	3.60E-08	-2.177	2.126	0.195	0.3060
rs987019	s__Romboutsia ilealis	0.076	0.013	T	0.166	8.40E-09	4.015	2.239	0.193	0.0733

SE: Standard error. EF: Effect allele. MAF: Minor Allele Frequency.

[1]: The association between the effect allele genetic variant (instrumental variable) and the exposure (Microbiome) was obtained from summary statistics published by Qin Y, et al. 2022 [51]. The effet allele for the exposure was harmonized to obtain a positive Beta (increasing effect in the exposure).

2: The second population was our Mediterranean population where the Beta was expressed for the same effect allele considered for the exposure.
